# Immunostimulatory effects of RACK1 pseudosubstrate in human leukocytes obtained from young and old donors

**DOI:** 10.18632/oncotarget.3002

**Published:** 2015-03-12

**Authors:** Emanuela Corsini, Valentina Galbiati, Antonella Pinto, Annalisa Davin, Letizia Polito, Antonio Guaita, Marco Racchi

**Affiliations:** ^1^ Laboratory of Toxicology, DiSFeB, Università degli Studi di Milano, Milan, Italy; ^2^ Department of Drug Sciences - Pharmacology, University of Pavia, Pavia, Italy; ^3^ “Golgi Cenci” Foundation, Abbiategrasso, Italy

**Keywords:** PKC, immunosenescence, cytokines, whole blood assay, signal transduction

## Abstract

Aims of this study were to investigate the ability of RACK1 pseudosubstrate alone or in combination with classical immune stimuli to activate human leukocytes, and to restore age-associated immune defects.

A total of 25 donors (17 old donors, 77–79 yrs; 8 young donors, 25–34 yrs) were enrolled. To evaluate the effect of RACK1 pseudosubstrate on cytokine production and CD86 expression the whole blood assay was used. Cultures were treated with RACK1 pseudosubstrate in the presence or absence of lipopolysaccharide (LPS) or phytohaemagglutinin (PHA) and incubated for 24 h or 48 h for LPS-induced CD86 expression, TNF-α, IL-6, IL-8, IL-10 production, and PHA-induced IL-4, IL-10, IFN-γ, respectively. RACK1 pseudosubstrate alone induced IL-6, IL-8, and CD86 expression in both young and old donors, and IFN-γ in old donors. In combination with LPS an increase in IL-8, IL-10 and TNF-α was observed, also resulting in restoration of age-associated defective production, while no changes in the other parameters investigated were found.

Even if based on a small sample size, these results suggest the possibility to by-pass some of age-associated immune alterations, which may be beneficial in situations were natural immune stimulation is required, and highlight a different role of PKCβ in immune cells activation.

## INTRODUCTION

Developed countries are experiencing an unprecedented increase in life expectancy that is accompanied by a rise in all age-associated problems, including immunosenescence and immuno-related disorders. Immunosenescence refers to the age-dependent changes responsible for the poor immune response to infections and the low efficacy of vaccination in elderly persons, which contributes to the enhanced rate of mortality and morbidity that are observed amongst the elderly [1]. Immunosenescence is characterized by a progressive deterioration of innate and adaptive immune functions, with immune cells displaying altered phenotype and function, thereby, providing an explanation for the clinical signs of immunosenescence [2–4].

In early studies aimed to characterize the molecular mechanisms underlying immunosenescence, we identified a defective protein kinase C (PKC) activation as a key player in the reduced response to immune stimulation. Specifically, we demonstrated that in the absence of a defect in total PKC expression, the failing element in its activation was the reduced expression of the Receptor for Activated C Kinase 1 (RACK1), which underlies functional impairment associated with aging, including cytokine production, cell proliferation [5–8], and response to influenza vaccination [9].

RACK1 is a 36-kDa protein that contains seven WD-domain motifs and is related to G protein β subunits [10, 11]. RACK1 is a highly conserved intracellular adaptor protein, which was originally identified as the anchoring protein for activated protein kinase C [12, 13]. In the past 20 years, the number of binding partners and validated cellular functions for RACK1 has increased, which is helping to define the versatile role of RACK1 in assembling and dismantling complex signaling pathways from the cell membrane to the nucleus in health and disease [14–16]. Relatively to PKC, RACK1 is able to interact preferentially with PKCβII [12] and PKCε [13], RACK1 modulates their activity by stabilizing their active conformation and promotes their translocation close to their specific substrates in order to activate defined pathways [10, 11]. PKCs play a key regulatory role in a variety of cellular functions, including cell growth and differentiation, gene expression, hormone secretion, etc. [17, 18]. Defective PKCβII translocation due to age-associated RACK1 decline was described in different immune cells [19, 20], in rat brain [21] and also in skin cells [22]. More recently, investigating the role of RACK1 and PKCβ in chemical allergen-induced CD86 expression and IL-8 production in the human promyelocytic cell line THP-1 and primary human dendritic cells, we demonstrated that a selective cell-permeable inhibitor of PKCβ completely prevented chemical allergen or LPS-induced CD86 expression and significantly modulated IL-8 production (50% reduction). Furthermore, the use of a commercially available RACK1 pseudosubstrate, consisting of a peptide derived from the C2 domain of PKCβ designed to directly activate PKCβ and linked by a disulfide bridge to the Antennapedia domain vector peptide for a rapid cell uptake, resulted in dose-related increase in CD86 expression and IL-8 production [23], supporting a model where PKCβ activation is a key component of the signal transduction pathways that induce activation of dendritic cells.

Based on the important role of RACK1 and PKC in cellular physiology and immune cells activation together with its role in immunosenescence, the purpose of this study was to investigate the ability of RACK1 pseudosubstrate alone or in combination with classical immune stimuli 1) to activate leukocytes obtained from healthy young and old donors and 2) to restore some of the age-associated immune defects. Results obtained demonstrate the possibility to by-pass some of the age-associated immune alterations by directly activating PKCβ, and overall highlight a different role of PKCβ activation in cytokine production.

## RESULTS

### Costimulatory effects of RACK-1 pseudosubstrate on LPS-induced THP-1 cells activation

We have recently demonstrated a role of PKCβ and RACK1 in allergen-induced CD86 expression and IL-8 production in both THP-1 cells and primary human dendritic cells, supporting a central role of PKC-β in the initiation of antigen-induced dendritic cells activation [23]. To further support these findings, and to investigate possible costimulatory effects of RACK1 pseudosubstrate, we assessed its ability alone or in combination with LPS to induce IL-6, IL-8 and TNF-α production and CD86 expression in THP-1 cells. As shown in Table 1, RACK1 pseudosubstrate alone was able to induce IL-8 production and CD86 expression, confirming previous results, while at the concentration tested (2.5 μM) and time investigated (24 h), RACK1 pseudosubstrate alone failed to induce IL-6 or TNF-α production, suggesting that selective activation of PKCβ is not sufficient for the production of these cytokines. To study possible costimulatory effects, a suboptimal concentration of LPS (10 ng/ml) was used together with RACK1 pseudosubstrate. As expected, LPS alone induced a significant increase in CD86 expression and IL-8 production, while a modest production of IL-6 and TNF-α was observed, indicative of a suboptimal concentration. The combination of LPS and RACK-1 pseudosubstrate was associated for all parameters measured, with stastically significant higher production compared to cells treated with LPS alone, indicative of a costimulatory effect.

**Table 1:** Costimulatory effect of pseudoRACK1 on LPS-induced CD86 expression and cytokine production in THP-1 cells

TREATMENT	CD86 (SI)	IL-6 (pg/ml)	IL-8 (pg/ml)	TNF (pg/ml)
Control	1	5 ± 1	20 ± 3	14 ± 19
LPS 10 ng/ml	3.13 ± 0.10**	23 ± 4	3744 ± 687**	53 ± 4
pseudoRACK1 2.5 μM	1.59 ± 0.19*	6 ± 1	60 ± 16*	6 ± 11
LPS + pseudoRACK1	4.47 ± 0.44**, §	180 ± 27**, §§	12713 ± 930**, §§	139 ± 40**, §§

10^6^/ml cells were treated with LPS, pseudoRACK1 or LPS + pseudoRACK1 for 24 h. Results are representative of three independent experiments. Means ± SD are shown, statistical analysis was performed with Tukey's multiple comparison test with *p < 0.05 and **p < 0.01 vs control, and §§*p* ≤ 0.01 vs cells treated with LPS alone.

These results *de facto* inspired the subsequent studies, the purpose of which was to evaluate *in vitro* the immunostimulatory effects of RACK1 pseudosubstrate using leukocytes obtained from young and old donors, with the ultimate goal of assessing the possibility to restore some of the immune deficiencies associated with aging.

### Effects of RACK1 pseudosubstrate and LPS on leukocytes obtained from young and old donors

A total of 25 healthy subjects, 17 elderly (77–79 years old, 7 women and 10 men) and 8 young (25–34 years old, 4 women and 4 men), were enrolled. Blood samples were taken on fasting early in the morning. In Table 2, the absolute and percentage count of leukocytes are reported. No statistically significant differences were observed among leukocytes betweeen young and old donors. This is important in light of the use of diluted whole-blood cultures. As all functional assays are carried out using the same amount of blood, it is essential to quantify both the relative and the absolute numbers of white blood cells. The use of whole blood may indeed mask some cytopenias or excess of a cell type, which may lead to false data interpretation.

**Table 2:** Absolute and differential count of leukocytes

GROUP	WBC (10^3^/μl)	NE (%)	LY (%)	MO (%)	EO (%)	BA (%)
Young (*n* = 8)	7.09 ± 1.81	51.96 ± 8.04	34.70 ± 7.47	8.80 ± 2.20	4.14 ± 2.26	0.42 ± 0.22
Old (*n* = 17)	6.47 ± 1.25	54.28 ± 8.35	32.79 ± 8.57	8.67 ± 2.24	4.05 ± 2.65	0.63 ± 0.27

Each value represents mean ± SD. No statistically significant differences were found among age groups. WBC, white blood cells; NE, neutrphils; LY, lymphocytes; MO, monocytes; EO, eosinophils; BA, basophils.

**Figure 1:**
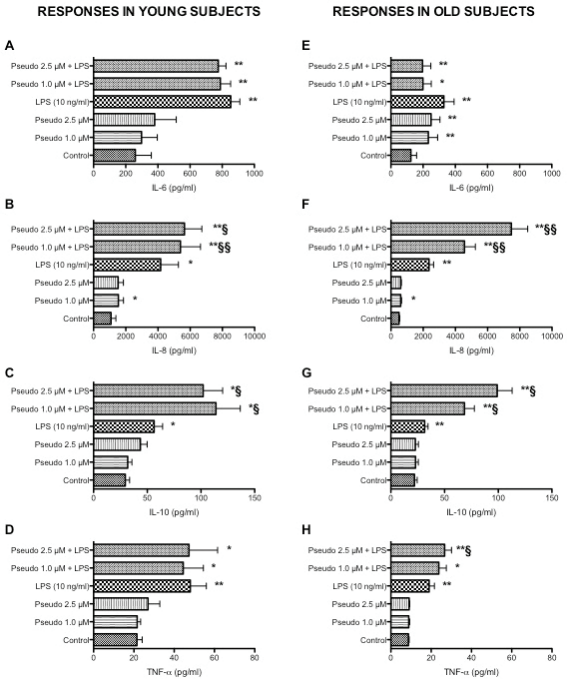
Effects of RACK1 pseudosubstrate alone or in combination with LPS on cytokine production in whole blood cultures 1:10 diluted whole blood cultures obtained from young (left graph, *n* = 8) and old donors (right graph, *n* = 17) were treated or not with two concentrations of RACK1 pseudosubstrate (1 and 2.5 μM) in the presence or absence of LPS (10 ng/ml) for 24 h. **(A, E)** Effect on IL-6 production. **(B, F)** Effect on IL-8 production. **(C, G)** Effect on IL-10 production. **(D, H)** Effect on TNF-α production. Results are expressed mean ± SEM. Statistical analysis was performed with pair Student's *t* test, with *p < 0.05 and **p < 0.01 vs control and §*p* < 0.05 and §§*p* < 0.01 vs LPS treated cultures.

**Figure 2:**
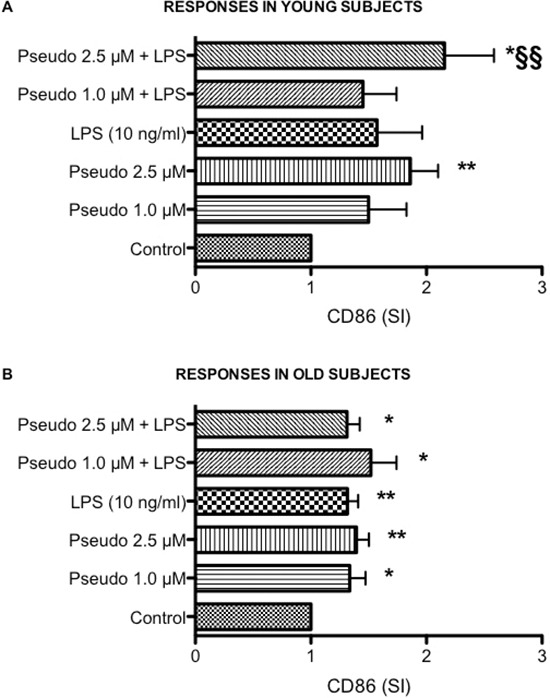
Effects of RACK1 pseudosubstrate alone or in combination with LPS on CD86 expression in whole blood cultures 1:10 diluted whole blood cultures obtained from young and old donors were treated or not with two concentrations of RACK1 pseudosubstrate (1 and 2.5 μM) in the presence or absence of LPS (10 ng/ml) for 24 h. **(A)** Effect on CD86 expression in young donors, *n* = 8. **(B)** Effect on CD86 expression in old donors, *n* = 17. Results are expressed mean ± SEM. Statistical analysis was performed with pair Student's *t* test, with *p < 0.05 and **p < 0.01 vs control and §*p* < 0.05 and §§*p* < 0.01 vs LPS treated cultures.

Whereas studies using purified peripheral blood mononuclear cells or cell lines have provided substantial insight on mechanism underlying activation and cytokine production, they may be limited in their scope because they do not include all cell-cell or cell-protein interactions that take place *in vivo*, the whole blood assay serves as a useful bridge between *in vivo* situation and isolated cells [28, 29]. The whole blood assay is an easy to perform *in vitro* test that closely mimics the human situation. It is faster and cheaper than the more established peripheral blood mononuclear cell based assay, require less blood and saves material since no isolation of cells is required [30, 31]. Consistently with our previous findings [7–9], aging was associated with a decrease in LPS-induced TNF-α production (Figure 1D and 1H, *p* < 0.01). In addition, a statistically significant reduced LPS-induced IL-6 (*p* < 0.01), IL-8 (*p* < 0.05) and IL-10 (*p* < 0.01) production was also observed in leukocytes obtained from old donors compared to young donors (Figure 1). Consistently with results obtained in THP-1 cells, RACK1 pseudosubstrate alone was able to induce IL-8 production (Figure 1B and 1F) and CD86 expression (Figure 2A and 2B) in both young and old donors. Contrary to THP-1 and young donors, in old donors RACK1 pseudosubstrate alone also induced a statistically significant production of IL-6 (Figure 1E). In combination with LPS, a costimulatory effect was observed for LPS-induced IL-8 (Figure 1B and 1F) and IL-10 (Figure 1C and 1G) production in both young and old donors, for TNF-α (Figure 1H) in old donors and CD86 expression in young donors (Figure 2A). No costimulatory effects were observed for LPS-induced IL-6 production (Figure 1A and 1E). Importantly, in old donors RACK1 pseudosubstrate was able to completely restore LPS-induced IL-8 (Figure 1F) and IL-10 (Figure 1G), and partially restore TNF-α (Figure 1H) production, which may be beneficial in situations were natural immune stimulation is required.

### Effects of RACK1 pseudosubstrate and PHA on leukocytes obtained from young and old donors

**Figure 3:**
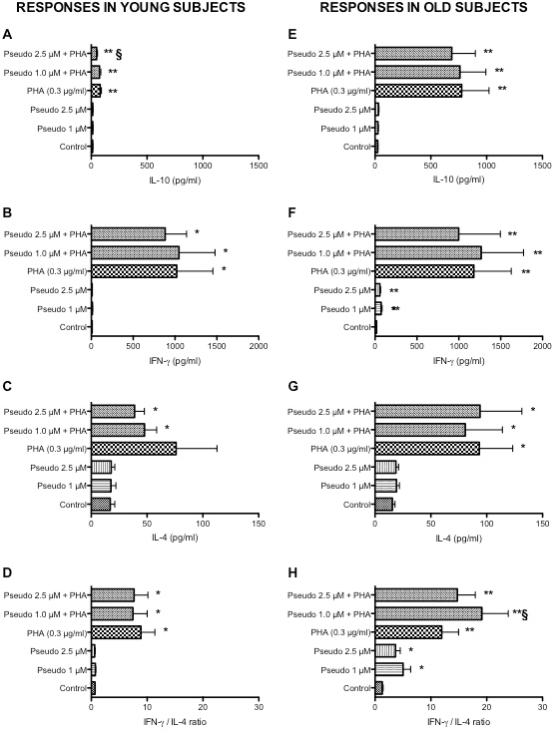
Effects of RACK1 pseudosubstrate alone or in combination with PHA on cytokine production in whole blood cultures 1:10 diluted whole blood cultures obtained from young (left graph, *n* = 8) and old donors (right graph, *n* = 17) were treated or not with two concentrations of RACK1 pseudosubstrate (1 and 2.5 μM) in the presence or absence of PHA (3 μg/ml) for 48 h. **(A, E)** Effect on IL-10 production. **(B, F)** Effect on IFN-γ production. **(C, G)** Effect on IL-4 production. **(D, H)** Effect on IFN-γ/IL-4 ratio. Results are expressed mean ± SEM. Statistical analysis was performed with pair Student's *t* test, with *p < 0.05 and **p < 0.01 vs control and §*p* < 0.05 and §§*p* < 0.01 vs LPS treated cultures.

In parallel, we explored the effect of RACK1 pseudosubstrate alone or in combination with PHA on IL-4, IL-10 and IFN-γ production (Figure 3). Diluted whole blood cultures were exposed for 48 h with RACK1 pseudosubstrate alone or in combination with PHA. Consistently with our previous findings [9], aging was associated with an increase in PHA-induced IL-10 production (Figure 3E, *p* < 0.01), while no change in IFN-γ was observed (Figure 3B and 3F). In addition to IL-10 and IFN-γ, we also measured the production of IL-4 and no changes in the production or in the IFN-γ/IL-4 ratio were observed in the two groups (Figure 3C, 3D and 3G, 3H). RACK1 pseudosubstrate alone had no effect on IL-4, IL-10 or IFN-γ production in leukocytes obtained from young donors (Figure 3A–3C), while a modest but statistically significant increase in IFN-γ production was observed in old donors at both concentrations tested (Figure 3F), which is also reflected in increased IFN-γ/IL-4 ratio in old donors (Figure 3H) compared to young donors (Figure 3D). In combination with PHA, the only relevant result was a significant reduction in the production of IL-10 in response to PHA in young donors (Figure 3A) and a similar trend in the elderly but not statistically significant (Figure 3B), together with a no dose-related increase in IFN-γ/IL-4 ratio in the old donors (Figure 3H). These results are indicative of a minor role of PKCβ in PHA-induced IL-4, IL-10 and IFN-γ production.

## DISCUSSION

The purpose of this study was to investigate the immunostimulatory effects of RACK1 pseudosubstrate in primary human leukocytes, with the ultimate goal of assessing the possibility to restore some of the immune defects associated with aging. Results showed that the direct activation of PKCβ using RACK1 pseudosubstrate, alone or in combination with classical immune activators, results in immune activation, as assessed by cytokine production, and in the revertion of some of the age-associated immune dysfuntions.

The concentrations of 1 and 2.5 μM of RACK1 pseudosubstrate were chosen from experiments in THP-1 cells, where a clear dose response was observed [23]. For some of the parameters measured, using primary leukocytes, we did not observe a clear dose response. In particular we observed a better or similar stimulation with 1 μM compared to 2.5 μM. We can speculate that this maybe due to a different kinetic in the production of cytokines in response to a different vigor in cell activation following RACK pseudosubstrate stimulation or to donor variability that may level the response, or to a saturation in the ability of RACK1 pseudosubstrate to activate PKC in fresh leukocytes compared to cell lines.

It is widely accepted that aging is characterized by a pro-inflammatory imbalance termed ‘inflammaging’, with increased serum levels of inflammatory cytokines (TNF-α, IL-1, IL-6), while increased anti-inflammatory cytokines (IL-10, TGF-β) are possibly associated with more healthy aging [32]. In a previous unpublished study conducted in the same geographical area, which includes some of the same donors enrolled in the current study, we found in the elderly group increased serum levels of IL-1β (1.8 ± 1.9 pg/ml vs 0.3 ± 1.0 pg/ml of the young group, *p* < 0.001. Results are expressed as mean ± SD, *n* = 20), TNF-α (23.9 ± 19.2 pg/ml vs 13.2 ± 42.1 pg/ml, *p* < 0.001) and IL-10 (3.1 ± 2.0 pg/ml vs 1.5 ± 2.9 pg/ml, *p* < 0.01), and decreased levels of IL-1RA (69.4 ± 65.9 pg/ml vs 210.0 ± 800.0 pg/ml, *p* < 0.05), consistent with literature data. In spite of this pro-inflammatory status, defective immune cells activation is observed when cells obtained from old donors are stimulated with classical immune activators [33]. In previous studies, using the whole blood assay, we found reduced TNF-α production in response to LPS, increased IL-10 and no change in IFN-γ production in response to PHA and decreased mitogen-induced T cell proliferation, which correlated with reduced leukocyte RACK1 expression [7–9]. No gender differences were observed. In the current study, the defective response to LPS was confirmed. Furthermore, not only TNF-α production in response to LPS was significantly reduced (*p* < 0.01) compared to young donors, but also IL-6, IL-8 and IL-10 were significantly reduced as well, indicating an overall reduction in monocyte activation. Such defective cytokine production in response to LPS, with the exception of IL-6, could be reversed by RACK1 pseudosubstrate. RACK1 pseudosubstrate directly activating PKCβ can by-pass the age-associated reduced RACK1 expression, and restore PKC-mediated cell activation. The costimulatory effect of RACK1 pseudosubstrate on LPS-induced IL-6 production observed in THP-1 cells could not be confirmed in primary cells obtained from both young and old donors. Overall, THP-1 cells are a good model to assess monocyte/macrophage functions, but they do not completely reflect the response of primary human cells. Concerning the activation of T cells, RACK1 pseudosubstrate alone had no effect on IL-4, IL-10 or IFN-γ production in leukocytes obtained from young donors, while a modest but statistically significant increase in IFN-γ production was observed in old donors. In combination with PHA, the only relevant result was a significant reduction in the production of IL-10 in response to PHA in young donors. A similar inhibitory effect on IL-10 production was also observed using in the young donors anti CD3/CD28 as stimuli (data not shown), no data are currently available on old donors. A slight reduction in IL-10 production was observed in the elderly, but it was not statistically significant. The concentration of PHA was selected using leukocytes obtained from young donors, in the case of IL-10, where old donors produce much higher levels, a lower concentration of PHA may be necessary to observe a modulation. Further studies are clearly necessary to characterize its specific role in T cell activation, including proliferation and T cell migration. Overall, these results are indicative of a minor role of PKCβ in PHA-induced IL-4, IL-10 and IFN-γ production.

It is well known that PKCs play a key regulatory role in a variety of cellular functions, including cell growth and differentiation, gene expression, hormone secretion, etc. [18–20, 34]; PKC family is the largest serine/threonine-specific kinase family known to comprise approximately 2% of the human kinome [35]. It is also clear that different PKCs are not functionally redundant, for example specific PKCs mediate specific cellular signals required for activation, proliferation, differentiation and survival of immune cells [36–39], and patterns of expression for each PKC isoform differ among tissues [18]. Of the classical PKCs isoforms, only PKC-βII was consistently activated during DC-differentiation-inducing stimuli in normal and leukemic progenitors [40]. PKC-βII activation by cytokines (GM-CSF + IL-4 + TNF-α) in DC was demonstrated to be associated to up-regulation of DC surface markers (MHCI and MHCII, CD11c, CD40, CD80, CD86 and CD83), the induction of expression of the NF-κB family member c-Rel, and the ability to stimulate allogeneic T cell proliferation. Our findings support a model where PKCβ activation is a key component of the signal transduction pathways that induce CD86 up-regulation and IL-8 production in monocyte/macrophage activation following exposure to LPS. On the contrary its role in T cell activation seems to be less prominent. This is consistent with data that highlight a central role of other PKC isoforms, namely PKCθ, PKCε and PKCα, in antigen receptor-mediated T cell activation *ex vivo* and T cell-mediated immunity *in vivo* [39]. In T cells, however, a role of PKCβ in T cell locomotion has been demonstrated, where T cells crowling has been shown to be associated with the translocation of PKCβ to the microtubule cytoskeleton [41]. Alterations in PKC expression and/or function may have important implications in health and disease and warrants a detailed investigation into the downstream target genes and the underlying mechanisms involved [42–45]. Further work is needed to identify mechanisms underlying PKCs recruitment or exclusion, potential redundancy, and relevance during immune cells activation.

With the limitation of being a small sample size study, nevertheless our findings contribute to the understanding of the mechanism underlying immunosenescence. The significant results obtained, although with the need of confirmation in larger cohort of subjects, open the possibility to use the selective activation of specific PKC isoforms to provide novel therapeutic strategies to manipulate monocyte activation and to counteract immunosenescence. As the effectiveness of vaccines is reduced in older versus younger adults because of age-related immunosenescence, the use of an adjuvant, such as RACK1 pseudosubstrate, may represent a strategy that may combat immunosenescence, potentially by boostering dendritic cells activation and T-cell mediated responses, resulting in a more effective response to vaccination. Development of drugs that target the PKCβ pathway may lead to novel therapeutic options for treating age-related disease including immunosenescence.

## METHODS

### Chemicals

Lipopolysaccharide from *Escherichia coli* serotype 0127:B8 was obtained from Sigma (St Louis, MO, USA); phytohaemagglutinin was from Invitrogen (PHA, Invitrogen, Paisley, UK). All reagents were purchased at the highest purity available.

### Experiments with THP-1 cells

THP-1 cells (Istituto Zooprofilattico di Brescia, Italy) were diluted to 10^6^ cells/mL in RPMI 1640 containing 2 mM L-glutamine, 0.1 mg/mL streptomycin, 100 IU/mL penicillin, 50 μM 2-mercaptoethanol, supplemented with 10% heated-inactivated foetal calf serum (media) and cultured at 37°C in 5% CO_2_ incubator.

To investigate the role of PKCβ in THP-1 activation, a pseudo RACK1 activator of protein kinase C was used (Tocris Bioscience, Bristol UK). The pseudosubstrate consists of a peptide derived from the C2 domain of PKC-β (24) linked by a disulfide bridge to the Antennapedia domain vector peptide. The sequence of the peptide, as reported from the supplier, is: KKWKMRRNQFWIKIQRC CSVEIWD* (Modifications: disulfide bridge between 17–1*). The Antennapedia peptide is actively taken up by intact cells, ensuring rapid and effective uptake of the activator peptide [25]. Once inside the cell, the disulfide bonds are subjected to reduction in the cytoplasm leading to production of the activator peptide. Cells were treated with concentrations and times specified in the legends.

To investigate the co-stimulatory effect of RACK1 pseudosubstrate, cells were treated simultaneously with the pseudosubstrate in the presence or absence of LPS (10 ng/ml).

### Study population

In this study a total of 25 healthy subjects living in the same geographical area of Northern Italy were recruited. These subjects included 17 elderly subject (age range 77–79; 7 women and 10 men) and 8 young subjects (age range 25–34, 4 women and 4 men). All elderly subjects were community dwelling, functionally independent and participating to the InVeCe Ab population based study [26]. Healthy subjects were selected according to the guidelines of the Italian Health authorities and to the Declaration of Helsinki principles and signed an informed consent. Criteria for exclusion were the presence of abnormal laboratory values (i.e. altered hemocrome) or the use of medication known to affect the immune system, i.e. steroids, or patients suffering from malignancies, inflammations and infections.

### Preparation of whole blood cells and cytokine production

Blood samples were taken by venous puncture with sodium citrate 0.5 M as anticoagulant. Sodium citrate was chosen instead of heparin or EDTA as anticoagulant, since functional assays were performed using the whole blood assay and heparin may be contaminated with endotoxin, while EDTA interferes with cell activation. Blood samples were diluted 1:10 in cell culture medium RPMI 1640 (Sigma, St Louis, USA) containing 2 mM L-glutamine, 0.1 mg/ml streptomycin, 100 IU/ml penicillin. Diluted blood samples were treated in the presence or absence of LPS at final concentration of 10 ng/ml or PHA 0.3 μg/ml and incubated for 24 h or 48 h at 37°C in a humidified 5% CO_2_ incubator for TNF-α, IL-6, IL-8, IL-10, and IL-4, IL-10 and IFN-γ production, respectively. Owing to the variability in stimulation assays, the same lot of each reagent was used in all experimental cultures. The suboptimal concentration of LPS and PHA were chosen in preliminary dose-response experiments. The use of suboptimal concentrations allow to highlight any costimulatory effects. Cell-free supernatants obtained by centrifugation at 1200 rpm for 5 min were stored at −20°C until measurement.

### Cytokine production

Cytokine production was assessed in cell free supernatants by a specific sandwich ELISA, commercially available (R&D System, Minneapolis, MN, USA). Results are expressed in pg/ml.

### Flow cytometric analysis of CD86 expression

CD86 expression was evaluated as previously described [27]. Briefly, after 24 h of treatment, THP-1 cells were centrifuged, washed once with cold PBS and suspended in PBS supplemented with 1% FCS and 0.1% NaN3. 10^5^ cells were stained in the dark for 30 min for THP-1 cells or 15 minutes for blood samples with specific FITC-conjugates antibodies against CD86 (BD Biosciences) or with isotype control antibody at room temperature (BD Biosciences). 1 ml of PBS was then added and cells centrifuged at 1200 rpm for 5 min and resuspended in 0.5 ml of PBS supplemented with 1% FCS and 0.1% NaN3. For the blood samples, after 24 h of treatment, dliluted whole blood was centrifuged at 1500 rpm for 5 min and pellets resuspended in 100 μl of PBS supplemented with 0.1% NaN3, 50 μl was stained in the dark for 15 minutes with the specific antibodies or with isotype control antibody at room temperature. 1 ml of FACS lysing solution (BD Biosciences) was then added and samples incubated for 10 min, then centrifuged at 1200 rpm for 10 min and resuspended in 0.5 ml of PBS supplemented with 0.1% NaN3. The intensity of fluorescence and the percentage of positive cells were analyzed using a FACSCalibur flow cytometer and data were quantified using CellQuest software (Becton Dickinson). 10,000 viable cells were analyzed for mean fluorescence intensity (MFI) and percentage of positive cells (PC). All experiments were performed in triplicate.

Changes in CD86 expression are reported as stimulation index (SI) calculated by the following equation:

S I=PC t×MFIt / PC c×MFIc

PCt and MFIt stand for treated cells, whereas PCc and MFIc for the untreated ones.

### Statistical analysis

All experiments using THP-1 were repeated at least three times, with representative results shown. Statistical analysis was performed using GraphPad InStat version 3.0a for Macintosh (GraphPad Software, San Diego, CA, USA). For multiple comparisons analysis of variance was used with Tukey post-hoc test. For blood samples, pair Student's *t* test was used. Differences were considered significant at *p* ≤ 0.05.

## Abbreviations

IL: interleukin
IFN: interferon
LPS: lipopolysaccharide
PHA: phytohaemagglutinin
RACK-1: receptor for activated C kinase-1
TNF: tumor necrosis factor
